# Cortical Development and Brain Malformations: Insights From the Differential Regulation of Early Events of DNA Replication

**DOI:** 10.3389/fcell.2019.00029

**Published:** 2019-03-11

**Authors:** Argyro Kalogeropoulou, Zoi Lygerou, Stavros Taraviras

**Affiliations:** ^1^Department of Physiology, Medical School, University of Patras, Patras, Greece; ^2^Department of General Biology, Medical School, University of Patras, Patras, Greece

**Keywords:** DNA replication, origins licensing, cortex development, neural stem cells, microcephaly

## Abstract

During the development of the cortex distinct populations of Neural Stem Cells (NSCs) are defined by differences in their cell cycle duration, self-renewal capacity and transcriptional profile. A key difference across the distinct populations of NSCs is the length of G1 phase, where the licensing of the DNA replication origins takes place by the assembly of a pre-replicative complex. Licensing of DNA replication is a process that is adapted accordingly to the cell cycle length of NSCs to secure the timed duplication of the genome. Moreover, DNA replication should be efficiently coordinated with ongoing transcription for the prevention of conflicts that would impede the progression of both processes, compromising the normal course of development. In the present review we discuss how the differential regulation of the licensing and initiation of DNA replication in different cortical NSCs populations is integrated with the properties of these stem cells populations. Moreover, we examine the implication of the initial steps of DNA replication in the pathogenetic mechanisms of neurodevelopmental defects and Zika virus-related microcephaly, highlighting the significance of the differential regulation of DNA replication during brain development.

## Introduction

The neocortex is a complicated brain region characterized by excessive cell diversity as it is composed by multiple types of neuronal and glial cells assembled in networks that regulate the higher order functions. The cortex derives from the dorsal telencephalon located in the most anterior part of the neural tube through a strictly regulated process that involves diverse types of neural stem cells (NSCs) and more committed neural progenitors (NPCs) organized in discrete zones (Azzarelli et al., 2015; Agirman et al., 2017). Excitatory neurons are produced consecutively within the proliferating zones and subsequently their somata migrate radially to their final position in the cortical plate. Cortical connectivity is completed with the integration of cortical interneurons that are generated in the ventral telencephalon and migrate tangentially toward the developing cortex (Dwyer et al., 2016; Lim et al., 2018).

Coordinated action of morphogens and intrinsic signaling cues leads to the successive generation of the distinct types of NSCs and NPCs during cortical development. These populations differ on their self-renewal ability and differentiation potential. Numerous studies have identified distinct morphological and molecular features that describe the diversity among NPCs (reviewed in Govindan and Jabaudon, 2017; Uzquiano et al., 2018).

DNA replication is a key cellular process that is coupled with growth and proliferation. Elucidating the differential regulation of DNA replication within the distinct populations of NPCs will expand our knowledge on their functional diversity. Interestingly, increasing evidence over the last decades supports the implication of defective DNA replication in the pathophysiology of cortical malformations like microcephaly (Jackson et al., 2014; Khetarpal et al., 2016). In this review, we will discuss the differential regulation of the initial steps of DNA replication, focusing on the NSCs of the early stages of cortical development and the impact of defective replication on normal development.

## Establishment of Neural Stem Cells During Early Cortical Development

**FIGURE 1:**
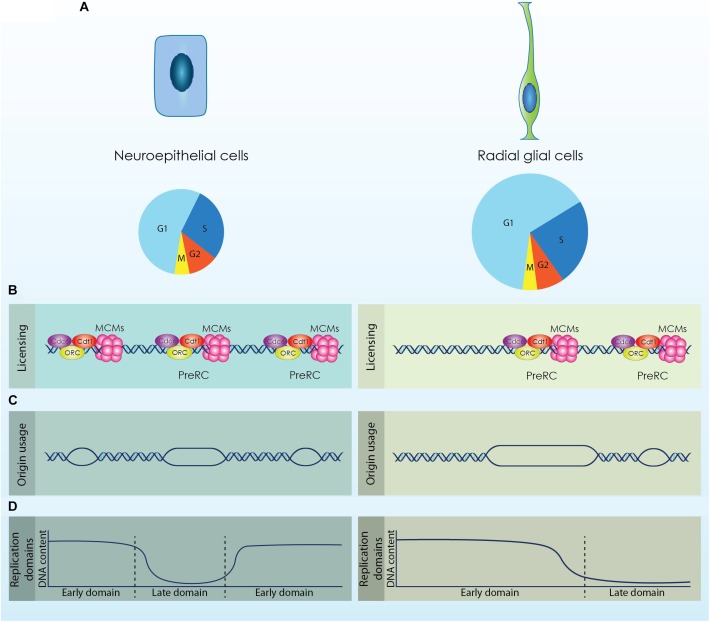
The diverse populations of neural stem cells (NSCs) that emerge during cortical development exhibit distinct cell cycle kinetics and differential regulation of the DNA replication licensing and initiation processes. **(A)** Neuroepithelial cells are characterized by a shorter cell cycle compared to Radial Glial cells that permits their fast proliferation. (**B**; Licensing) During the G1 phase origins of replication are licensed by the formation of a competent pre-replicative complex (Pre-RC). Higher expression of licensing factors (ORC, Cdc6, Cdt1 and MCMs) is required for the efficient licensing of origins in NECs that exhibit a shorter G1 phase. (**C**; Origin usage) Increased formation of Pre-RCs facilitates the usage of more origins for the accurate completion of DNA replication that further causes a reduction of the available dormant origins. RGs activate less origins of replication compared to NECs to complete the duplication of their genome. (**D**; Replication Domains) DNA is replicated in well-defined segments constituted by multiple origins that fire synchronously in distinct time points throughout S phase. Rapid proliferating cells complete DNA replication in smaller segments, while cells with longer G1 phase exhibit fewer but larger replication domains.

The first population of NSCs that is established in the developing cortex are the neuroepithelial cells (NECs), while at the onset of neurogenesis NECs are replaced by apical radial glial cells (aRGs) that remain in contact with the apical membrane and form the ventricular zone (VZ), one of the main proliferating zones of the cortex. During mid neurogenesis a second proliferating zone is established basally of the VZ by intermediate progenitors, which delaminate from the apical membrane and are translocated to the subventricular zone (SVZ). Despite these primary populations, other minor groups of progenitors are also generated over the course of development like the short neural precursors (SNPs) that appear in the VZ and the outer RGs (oRGs) located in the upper boundaries of the SVZ. Here, we will focus on the populations of the apical NSCs that prevail the VZ during the initial stages of cortical generation (Figure 1A). A more detailed description of the NSCs and NPCs population of the developing cortex are reviewed by Götz and Huttner (2005), Taverna et al. (2014), and Uzquiano et al. (2018).

Neuroepithelial cells consist the first population that reside in the walls of the neural tube. These cells are highly polarized, possessing a basal process that is attached to the basal lamina and an apical process attached to the apical surface in contact with the neural tube lumen (Arai and Taverna, 2017). NECs sense and respond to signaling cues from the cerebrospinal fluid through a primary cilium that protrudes from their apical membrane into the lumen (Guemez-Gamboa et al., 2014). The nuclei of NECs move across the apical-basal distance in a cell cycle dependent way; mitosis occurs always in the apical surface of the neural tube and as the cell progresses to the G1 phase the nucleus is moving radially toward the basal side. During DNA replication the nucleus reaches the basal membrane and migrates to the opposite direction until it will be repositioned to the apical surface for a new division. This process is known as interkinetic nuclear migration (INM) and gives to the neuroepithelium the appearance of a pseudostratified epithelium (Miyata et al., 2014). NECs exhibit a relatively short cell cycle (∼12 h) that correlates with the high proliferation potential of these cells and permits the establishment of the initial pool of NSCs through symmetric self-renewing divisions (Takahashi et al., 1995; Borrell and Calegari, 2014).

Upon the onset of neurogenesis at embryonic day (E)10.5 in murine development and until E12.5, NECs are gradually transformed to aRGs, which constitute the main population of NSCs during cortical development. aRGs are also polarized cells exhibiting an apical and a basal process that span the radial axis of the developing cortex and maintain their primary cilium that projects into the newly formed telencephalic vesicles (Arai and Taverna, 2017). Similar to NECs, aRGs undergo INM during a cell cycle but the movement of their nucleus is restricted within the VZ (Miyata et al., 2014; Bertipaglia et al., 2017). SNPs that constitute a subtype of aRGs also appear in the VZ during that stage. SNPs remain in contact with the apical surface of the cortex through their apical process, however, their basal process does not span until the basal membrane but is constrained to the VZ (Stancik et al., 2010). The transition of NECs to aRGs is critical during brain development as it defines the switch from a self-renewing to a neurogenic state. The establishment of aRGs is defined by the elongation of the cell cycle (∼17 h) owed primarily to an increase of the G1 phase length, which doubles during mid-neurogenesis and covers more than 60% of aRGs cell cycle (Noctor et al., 2002; Calegari et al., 2005). Elongation of the G1 phase allows the action of fate determinants and the respective response of the cells (Matsuzaki and Shitamukai, 2015).

During cortical development, extrinsic signaling cues derived from the local environment coordinate the proliferation and fate commitment of NSCs. Various fate determinants and signaling pathways impact not only in the genetic program of NSCs by regulating the expression of specific factors, but also in their intrinsic features like their cell cycle and chromatin dynamics (Borrell and Calegari, 2014; Albert et al., 2017). Activation of the Notch pathway was identified to mediate NECs to aRGs transition, when an activated form of the Notch1 receptor was introduced in the telencephalon of E9.5 embryos and promoted radial glia identity (Gaiano et al., 2000). Upon establishment of aRGs, Notch signaling promotes cell cycle progression and maintenance of this population (Ohtsuka et al., 2001). Notably, the fibroblast growth factor (FGF) signaling pathway has key roles during aRGs specification and maintenance and thus controls neuronal output and proper cortical formation (Sahara and O’Leary, 2009; Rash et al., 2013). Interestingly, it has been shown that FGF signaling promotes proliferation of NPCs by directly decreasing the G1 length, suggesting an interplay between fate determinants and cell cycle regulation (Lukaszewicz et al., 2002).

## An Overview of DNA Replication Licensing and Initiation Steps in Eukaryotic Cells

Within a complete cell cycle the genetic information must be faithfully duplicated and pass intact to the progeny. Moreover, in the level of multicellular organisms, replication must be coordinated with cell fate decisions that are mediated by complicated transcriptional programs. DNA replication is organized at multiple levels to ensure that genome duplication will be completed within the available time and over- or under- duplication that threatens genome integrity will be avoided. In eukaryotic cells, DNA replication initiates from multiple sites along the genome known as replication origins. Initiation of replication is strictly regulated by a two-step process that involves the formation of a multiprotein complex termed pre-replicative complex (Pre-RC) on the potential origins and is called origins’ licensing, followed by the sequential activation of a subset of origins known as origins’ firing (reviewed in Symeonidou et al., 2012; Fragkos et al., 2015).

During the G1 phase replication origins are licensed by the sequential recruitment of proteins that will form an inactive pre-RC (Figure 1B; Licensing). The Origin Recognition Complex (ORC1-6) is the first factor that recognizes and binds to the origins serving as a docking site for the association of the rest of the licensing proteins. Following, the licensing factors Cdc6 and Cdt1 interact with the bound ORCs and promote the loading of the Minichromosome Maintenance complex (MCM) resulting in a competent pre-RC (Champeris Tsaniras et al., 2014). The MCM complex consists of six subunits (MCM2 to MCM7) with an ATP dependent DNA helicase activity and is loaded to chromatin as an inactive head-to-head double hexameric ring that encircles double-stranded DNA. To ensure the timed duplication of the genome, cells license more origins than the origins that require and therefore load more MCM complexes into chromatin, as reduced MCM loading makes the cells sensitive to replication stress (Ibarra et al., 2008). Firing of origins occurs during the G1/S transition with the binding of the CDC45 and GINS to MCM2-7 that will form a stable CMG (CDC45-MCMs-GINS) complex with helicase activity. From that point, multiple replication factors are recruited to the activated origins to form the replisome, which is consisted of an active helicase subunit and DNA polymerases and is moving along the DNA to complete replication (Sun et al., 2015).

Upon entry to S phase multiple mechanisms negatively regulate licensing by phosphorylating the licensing factors and thus preventing the re-assembly of the Pre-RC (reviewed in Siddiqui et al., 2013). Phosphorylated CDT1 is targeted for degradation by the E3 ubiquitin ligase SCF-Skp2, while the chromatin-bound fraction of CDT1 is ubiquitinated by the CRL4-Cdt2 (Li et al., 2003; Nishitani et al., 2006). In metazoan an additional mechanism for CDT1 regulation is mediated by Geminin. During the S, G2 and M phases of the cell cycle Geminin binds to CDT1 and thus prevents its association with other licensing factors. Once mitotis is completed Geminin is deactivated permitting a new round of origins licensing (Caillat and Perrakis, 2012). CDC6 is also phosphorylated upon its release from chromatin and is translocated to the cytoplasm (Delmolino et al., 2001). Finally, in mammalian cells, the ORC1 subunit is specifically dissociated from chromatin due to ubiquitination and part of it is degraded (Li and DePamphilis, 2002).

## Regulation of the Licensing Factors During the Short G1 Phase of NECs

Neuroepithelial cells are characterized by an unusual cell cycle structure compared to the populations of neural stem and progenitor cells that are generated later during the development of the cortex. NECs exhibit a relatively small cell cycle while the length of the G1 phase is gradually increased as these cells are transformed to a RGs (Takahashi et al., 1995; Calegari et al., 2005). The short cell cycle length ensures the fast proliferation of NECs and the establishment of an initial pool of NSCs within a defined period during embryogenesis. Therefore, origin licensing during DNA replication must be accurately completed in the restricted cell cycle duration of NECs in order to secure faithful duplication of the genome. Consequently, a question that is raised is whether NECs employ different mechanisms to regulate licensing of DNA replication than other populations of NSCs or NPCs.

Neuroepithelial cells exhibit comparable cell cycle kinetics with Embryonic stem cells (ESCs), as both populations have similar cell cycle duration. It is also established that a short G1 phase is an intrinsic property of multipotency as both in ESCs and NECs G1 length increases upon fate commitment (Calegari et al., 2005; White and Dalton, 2005). The potential to *in vitro* differentiate ESCs to NPCs offers a suitable system for monitoring modifications in the licensing of DNA replication along with the progressive elongation of the cell cycle and neural fate commitment. It has been shown that ESCs express and maintain higher levels of CDT1 and CDC6 compared to differentiated cells to secure sufficient licensing and timed initiation of DNA replication (Fujii-Yamamoto et al., 2005; Ballabeni et al., 2011). Moreover, increased expression of licensing factors in hESCs mediates rapid MCM loading to chromatin, which facilitates the licensing of a “sufficient” number of origins within their short G1 phase. Interestingly, neuronal differentiation entailed with reduced expression of licensing factors and G1 elongation was sufficient to reduce the loading rate of MCM proteins (Matson et al., 2017).

These observations suggest that NECs might also require similar adaptations in the licensing of DNA replication due to their shortened G1 phase, while these features are probably absent from more committed NPCs defined by a longer G1 (Figure 1B; Licensing). Analyses of NPCs derived from different developmental stages are required to establish the differential regulation of licensing.

## Balance Between Origins Usage and Dormant Origins Contributes to Cortical Integrity

Eukaryotic cells license a greater number of origins during G1 phase compared to the origins that will fire to complete genome duplication. Some of the licensed origins that are not activated remain dormant and fire to cover unreplicated regions when the progression of the initially formed replication forks is impeded. Interestingly, reduction of dormant origins (DOs) challenges the successful completion of DNA replication compromising genome stability (Alver et al., 2014; Shima and Pederson, 2017). NPCs that carry the hypomorphic allele MCM4^chaos^, revealed significant decrease in DOs and exhibit increased DNA damage and reduced proliferation *in vitro*. Interestingly, reduction in DOs had also a direct effect in brain development, as embryos homozygous for the MCM4^chaos^ allele showed a thinner cortex during mid-embryogenesis due to increased cell death of intermediate NPCs resulted in defective generation of neurons (Ge et al., 2015). Accordingly, deletion of the MCM2 subunit using an inducible CreERT2 system resulted in impaired neurogenesis in the adult brain caused by a significant decrease in NSCs of the SVZ (Pruitt et al., 2007). It thus appears that reduction in the number of licensed origins that eliminates the abundance of DOs, leads to DNA damage probably because of unresolved replication structures and causes eventually the apoptosis of NSCs.

During the expansion phase of the developing cortex, NSCs utilize a high number of origins during DNA replication due to their short cell cycle (Ge et al., 2015). However, this inherent mechanism sensitizes NSCs against exogenous stress as it restricts the available DOs that could be activated as a response to stress. As the cell cycle length of NSCs is progressively increased, more DOs are available and NSCs become less sensitive to genotoxic stress. It can be therefore speculated that there is a threshold of available DOs to safeguard accurate genome duplication of NSCs (Figure 1C; Origin usage). Additional data are required to establish this elegant mechanism that safeguards genomic stability of cortical NSCs and secures structural and functional integrity of the cortex.

## Replication Timing Modifications Permit Rapid Adaptation to Developmental Cues During Cortical Development

In eukaryotic cells initiation of DNA replication is dispersed throughout the S phase resulting in chromosomal segments that are replicated in defined points, known as replication domains (Rivera-Mulia and Gilbert, 2016; Takebayashi et al., 2017). Replication timing of most domains is conserved among different cell types, however, specific domains that are subjected to developmental control have been also identified. Comprehensive analyses of *in vitro* systems that recapitulate the progressive fate commitment of mouse and human ESCs confirmed the relation between replication timing and gene expression and showed that changes in the timing of replication coordinate with transcriptional activation (Hiratani et al., 2010;Rivera-Mulia et al., 2015).

Upon commitment of ESCs toward the neuronal lineage, the 20% of the genome is subjected to replication timing modifications. These modifications include mainly consolidation of replication domains that lead to fewer but larger segments of coordinated replication (Figure 1D; Replication domains) (Hiratani et al., 2008). Coordination between transcription and replication is critical as conflicts between the two machineries would lead to defective gene expression and moreover to genomic instability (Lin and Pasero, 2012; García-Muse and Aguilera, 2016). During cortical development, NSCs are subjected to a strict developmental program that defines their transcriptional profile. Completion of DNA replication in larger segments permits the rapid adaptation of the transcriptional program by minimizing the possibility of collisions (Figure 1B; Replication domains). Thus, dynamic regulation of replication timing in NSCs is critical for an effective response to the solid developmental program that is required during cortex formation.

## Impaired Regulation of DNA Replication Results in Brain Malformations

Genetic or environmental factors that limit the proliferation potential of stem or progenitor cells during embryogenesis result in a variety of developmental abnormalities in human (Faheem et al., 2015; Ernst, 2016). Perturbed regulation of DNA replication, leading to a significant decrease in proliferating cells, has been already associated not only with developmental retardation but also with brain malformations like microcephaly (Table 1) (Mazouzi et al., 2014; Khetarpal et al., 2016). The distinctive features of NSCs regarding the licensing and initiation of DNA replication are critical for their rapid proliferation, required during the initial stages of brain development, highlighting the sensitivity of the brain to defected DNA replication.

**Table 1:** Genes linked to microcephaly that encode proteins involved in DNA replication.

Disease	Phenotype	Genes	Functions	Reference
Meier-Gorlin Syndrome	Microcephaly, primordial dwarfism and hypoplastic patella	*ORC1, ORC4, ORC6, CDT1* and *CDC6*	Pre-Replicative Complex formation	de Munnik et al., 2015
		Geminin	Re-licensing inhibitor	Burrage et al., 2015
		*CDC45, MCM5*	CMG complex establishment	Fenwick et al., 2016; Vetro et al., 2017
Bloom syndrome	Microcephaly, short stature and cancer predisposition	*BLM*	Replication fork stability and resolution of replication intermediates	German et al., 2007; Guo et al., 2007
Seckel syndrome (ATR/ATRIP-related)	Microcephaly, primordial dwarfism and dysmorphic facial features	*ATR*, ATR-Interacting Protein	Response to replication stress and stabilization of stalled forks	Goodship et al., 2000; Ogi et al., 2012; Mokrani-Benhelli et al., 2013
Microcephalic Primordial Dwarfism	Microcephaly and primordial dwarfism	*DONSON*	Replication fork stability	Reynolds et al., 2017


Meier-Gorlin Syndrome (MGS) is of the main syndromes that has set the link between licensing of DNA replication and brain development. MGS is an autosomal recessive type of primordial dwarfism due to developmental retardation, characterized by proportionate growth deficits, incomplete patellae formation and typical facial features (de Munnik et al., 2015). Severity of the symptoms varies across MGS patients and, interestingly, most severe cases are additionally diagnosed with microcephaly (Bicknell et al., 2011b; Burrage et al., 2015). Genetic analyses on reported cases of MGS revealed that biallelic mutations in the genes that form the pre-RC (ORC1, ORC4, ORC6, CDT1 and CDC6) are implicated in the syndrome (Bicknell et al., 2011a; de Munnik et al., 2012). *De novo* mutations in the licensing inhibitor Geminin were also identified in a group of patients (Burrage et al., 2015). The implication of DNA replication in MGS was further supported when, recently, mutations in the genes of CDC45 and MCM5 that form an active CMG complex were associated with the syndrome (Fenwick et al., 2016; Vetro et al., 2017). Studies performed in patient derived cell lines indicate delayed entry to S phase and decreased proliferation caused by insufficient licensing of DNA replication (Bicknell et al., 2011b; Burrage et al., 2015). Similar results indicating reduced proliferation were also obtained when mutated alleles previously identified in MGS patients were studied in heterologous systems (Guernsey et al., 2011).

**FIGURE 2:**
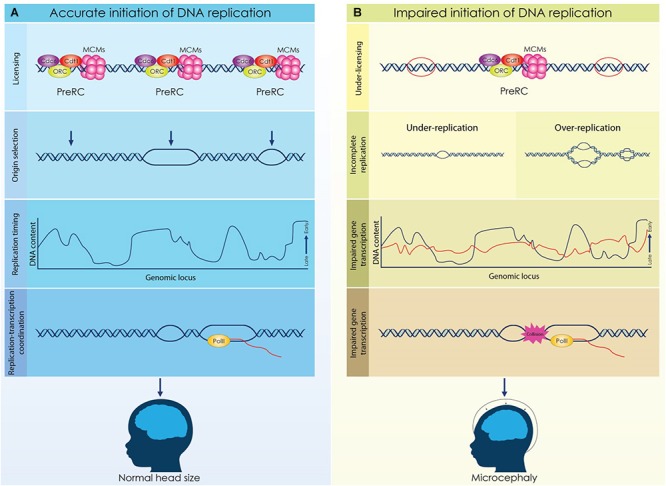
Licensing, origin selection and replication timing are dynamically regulated during cortical development to secure the formation of a functional cortex. **(A)** Origins of replication are licensed during the G1 phase by the formation of a competent pre-replicative complex (Pre-RC). A fraction of the licensed origins is selected during the S phase to establish a bidirectional replication fork while not activated origins remain dormant. DNA is replicated in well-defined chromosomal segments, that are duplicated in distinct time points throughout S phase. Establishment of a characteristic replication timing profile permits the coordination of replication and transcriptional machineries. **(B)** Impaired licensing and initiation of DNA replication lead to defective brain development and microcephaly. Reduced expression of licensing factors results in decrease of licensed origins that further causes incomplete initiation of DNA replication. Aberrant licensing directly affects the successful duplication of the genome due to under-replication or over-replication. Moreover, deviation of the normal replication timing due to defective licensing, can lead to impaired gene expression and possibly, to conflicts between replication and transcription. Under these conditions, the proliferation of NSCs is compromised resulting in severe brain malformations.

The microcephaly observed in MGS patients emphasizes the role of proper licensing of DNA replication during brain development (Figure 2). It is evident that impaired licensing severely compromises the rapid proliferation of NSCs resulting in insufficient generation of the initial NSCs pool and eventually in incomplete development. However, the mechanism that underlies the decreased proliferation of NSCs in MGS remains elusive. Proliferating NSCs accumulate higher levels of licensing factors due to their shorter G1 phase, consequently, in the case of the MGS where the expression of these factors is reduced, decreased formation of Pre-RCs and under licensing occur. Given that proliferating NSCs utilize more replication origins to complete genome duplication, under licensing has a direct effect on the progression of the S phase. Alternatively, the reduction of NSCs that is observed in MGS patients could be appointed to defected gene expression. Dynamic regulation of the replication timing in NSCs ensures the implementation of the respective transcriptional program, thus discordant DNA replication due to impaired licensing could affect normal gene expression resulting in brain malformations.

Environmental factors may further affect the proliferation of NSCs. Infectious agents that target NSCs, disrupt the early stages of cortical development resulting in neurological phenotypes. Due to its recent outbreak, Zika virus (ZIKV) is one of the most known aetiologies of infectious microcephaly, however, congenital infections including the cytomegalovirus or the parasite *Toxoplasma gondii* have been also associated with microcephaly (Devakumar et al., 2018). The implication of ZIKV in congenital microcephaly was established when its RNA was detected in the brain tissues of microcephalic fetuses as well as in the amniotic fluid of their infected mothers (Driggers et al., 2016). Initial studies on hiPSCs derived NPCs and brain organoids showed that proliferating NPCs are the main population of cells targeted by ZIKV, while differentiated neurons are mildly affected (Garcez et al., 2016; Tang et al., 2016). Interestingly, analysis of the cell cycle profiles showed that infected NPCs are accumulated to the S phase resulting in reduced proliferation and eventually apoptosis (Tang et al., 2016). Moreover, the brains from established animal models for ZIKV infection also exhibited a decrease in proliferating NPCs due to altered cell cycle kinetics (Li et al., 2016; Wu et al., 2016). In line with these results, transcriptomics analysis of infected NPCs confirmed the downregulation of genes involved in cell cycle and specifically DNA replication progression (Zhang et al., 2016). The possible effects of ZKV in the normal progression of DNA replication is a very intriguing scenario that could explain the preference of the virus toward proliferating NSCs.

## Conclusion and Perspectives

During the last decade the importance of the dynamic regulation of DNA replication during organismal development has been established (Nordman and Orr-Weaver, 2012; Champeris Tsaniras et al., 2014; Hua and Orr-Weaver, 2017). In the present review we describe differences in the processes of initiation of DNA replication between different populations of NSCs. NECs exhibit a short cell cycle with a short G1 phase similar to ESCs. For that reason they will require increased levels of licensing factors to facilitate the higher number of licensed origins required in order to cope with their accelerated cell cycle. Consequently, the number of the available DOs is reduced in rapid proliferating NECs increasing the risk of replication stress and genotoxic insults that would compromise the survival of NSCs. More data regarding the formation of licensing and initiation of DNA replication in NECs and RGs are needed to establish the above hypothesis which is mainly based on our knowledge on ESCs.

Moreover, adaptations in the number of licensed origins and in the replication timing profile permit the integration of the active transcriptional program that operates in NSCs during cortical development. How the plasticity of DNA replication initiation is regulated within the complicated environment of the developing brain and whether it is determined by signaling cues or it is an inherent feature of NSCs remain important questions.

Mutations in the genes that express licensing factors have been associated with developmental retardation characterized by microcephaly, stressing the significance of efficient licensing and accurate initiation of DNA replication during brain development. A future direction will be the establishment of animal models for impaired-licensing syndromes, that will permit the detailed analysis of brain development under these conditions. Further insights into the response of NSCs upon DNA replication stress will be critical to our understanding of the pathophysiology of neurodevelopmental syndromes. Accordingly, the possible effects of ZKV in the normal progression of DNA replication is a very intriguing scenario that could explain the preference of the virus in early proliferating NSCs. Deciphering the role of the differential regulation of DNA replication will provide new grounds for research on the mechanisms leading to brain malformations like the hereditary orinfectious microcephaly.

## Author Contributions

AK provided the study material and wrote the manuscript. ZL performed proofreading of the manuscript and approved final version of the manuscript. ST wrote the manuscript, approved final version of the manuscript, and provided financial support.

## Conflict of Interest Statement

The authors declare that the research was conducted in the absence of any commercial or financial relationships that could be construed as a potential conflict of interest.
